# Supplementary material for: Replication-attenuated r3LCMV vectors potentiate tumor control via IFN-I
Source: bioRxiv. 2023 Dec 8:2023.12.08.570847. Preprint. [Version 1] doi: 10.1101/2023.12.08.570847 (PMC10723415; doi:10.1101/2023.12.08.570847)
Supplement: Supplement 1 [file NIHPP2023.12.08.570847v1-supplement-1.pdf]

bioRxiv preprint doi: https://doi.org/10.1101/2023.12.08.570847; this version posted December 8, 2023. The copyright holder for this preprint (which was not certified by peer review) is the author/funder, who has granted bioRxiv a license to display the preprint in perpetuity. It is made available under aCC-BY 4.0 International license.

Supplemental Figure Legends:

Figure S1

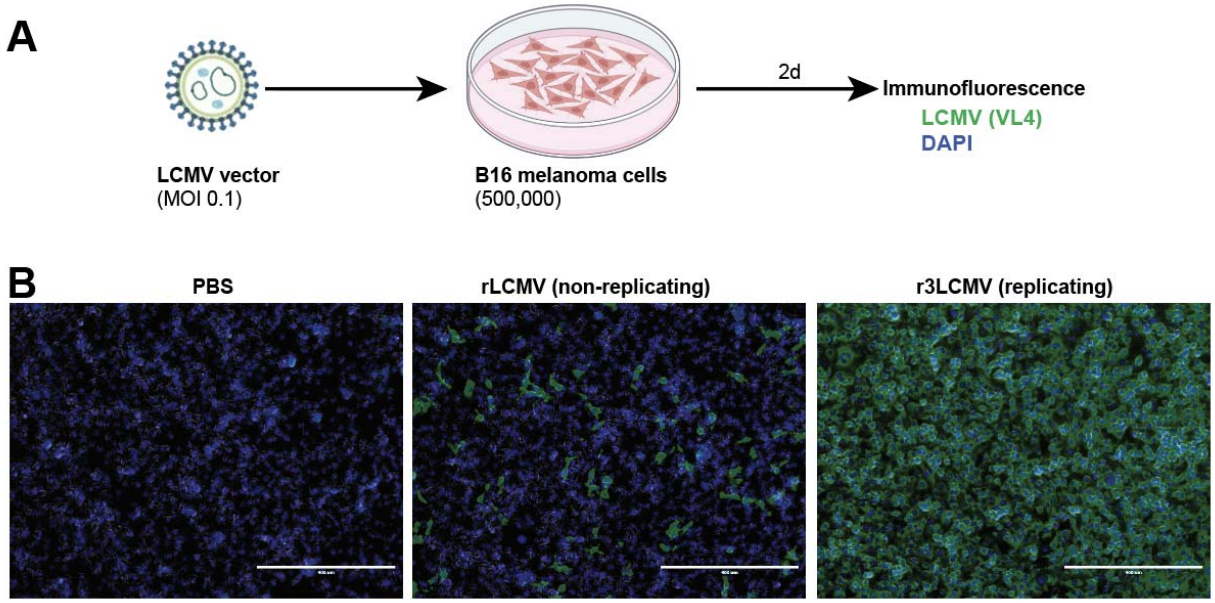

Figure S1. Attenuated r3LCMV replicates in B16 melanoma cells. (**A**) Experiment outline for detecting viral antigen after in vitro infection of B16 melanoma cells with replicating (r3LCMV) or non-replicating (rLCMV) vectors. (**B**) Representative immunofluorescence staining in B16 monolayers at day 4 post-infection. In this experiment, we detected substantially more viral antigen in B16 monolayers that were infected with replicating (r3LCMV), relative to non-replicating (rLCMV) vector. Experiment was performed 2 times with similar results.

bioRxiv preprint doi: https://doi.org/10.1101/2023.12.08.570847; this version posted December 8, 2023. The copyright holder for this preprint (which was not certified by peer review) is the author/funder, who has granted bioRxiv a license to display the preprint in perpetuity. It is made available under aCC-BY 4.0 International license.

Figure S2

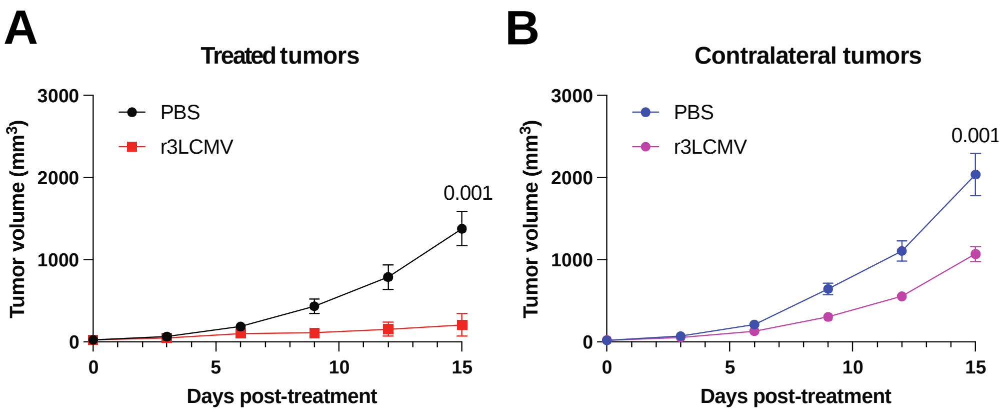

Figure S2. Attenuated r3LCMV induces abscopal effect. Mice were treated intratumorally with $2x10^5$ PFU of r3LCMV on the left tumor, five days after bilateral tumor challenge. (**A**) Tumor control on the treated tumor (left side). (**B**) Tumor control on the contralateral untreated tumor (right side). Data are from 2 experiments with a total of n=9-10 per group. Error bar represents SEM. Indicated P values were calculated by the Mann–Whitney test.

bioRxiv preprint doi: https://doi.org/10.1101/2023.12.08.570847; this version posted December 8, 2023. The copyright holder for this preprint (which was not certified by peer review) is the author/funder, who has granted bioRxiv a license to display the preprint in perpetuity. It is made available under aCC-BY 4.0 International license.

Figure S3

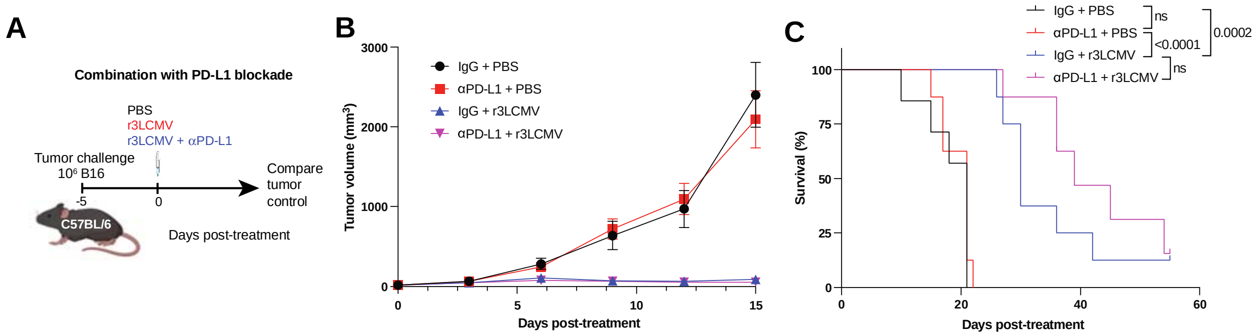

Figure S3. Effect of combined PD-L1 blockade and r3LCMV therapy. (**A**) Experiment outline for evaluating whether PD-L1 blockade improves r3LCMV therapy. (**B**)Tumor control. (**C**) Survival. Data are from 2 experiments with a total of n=6-7 per group. Error bar represents SEM. Indicated P values were calculated by the Kruskal-Wallis test and Dunn's multiple comparison test, or Kaplan-Meier when comparing survival.

bioRxiv preprint doi: https://doi.org/10.1101/2023.12.08.570847; this version posted December 8, 2023. The copyright holder for this preprint (which was not certified by peer review) is the author/funder, who has granted bioRxiv a license to display the preprint in perpetuity. It is made available under aCC-BY 4.0 International license.

Figure S4

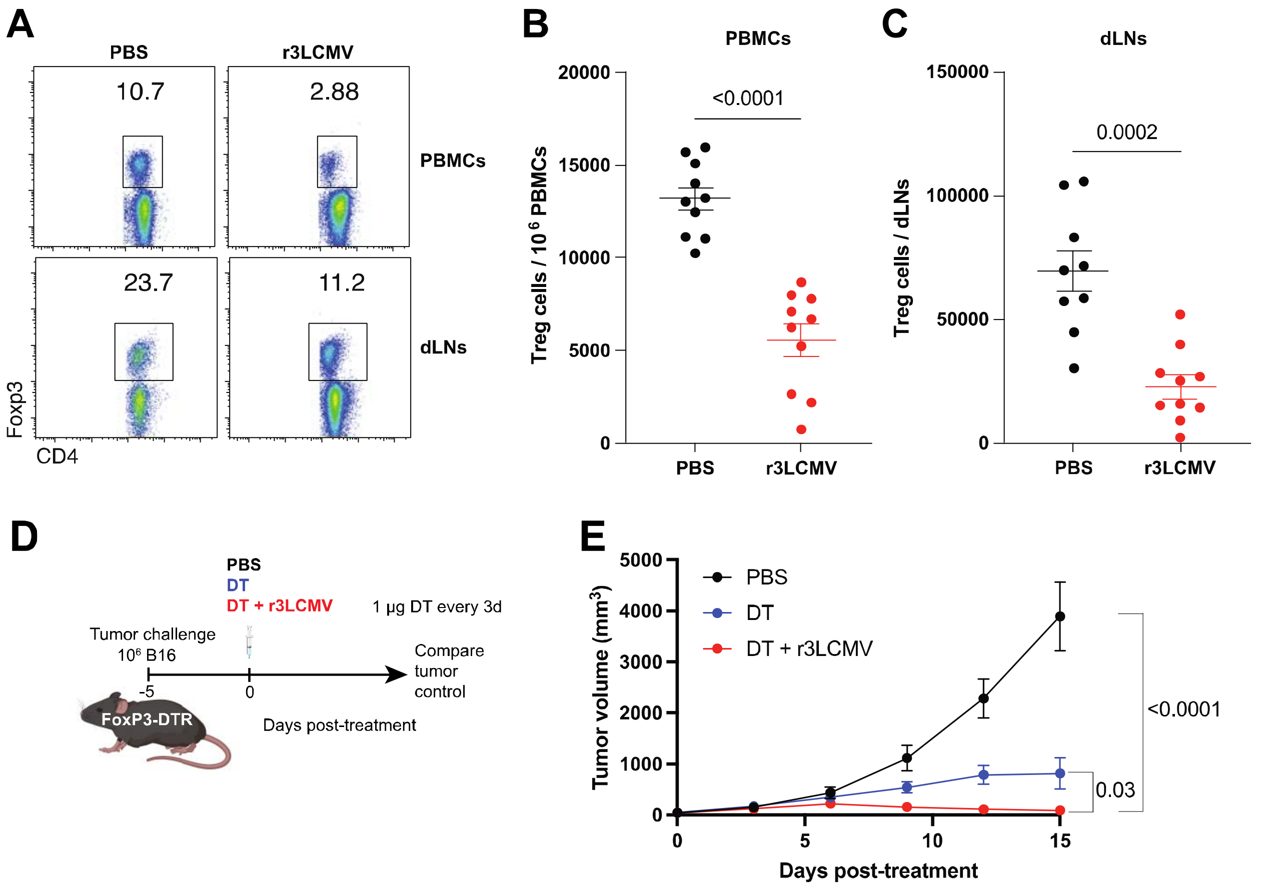

Figure S4. r3LCMV therapy results in a decline in Tregs. (**A**) Representative FACS plots showing Treg cell responses (gated on live CD4 T cells). (**B**) Summary of Treg cell responses in PBMCs. (**C**) Summary of Treg cell responses in tumor draining lymph nodes. Data from PBMCs are from day 7 post-treatment, and data from tumor draining lymph nodes are from day 8 post-treatment. (**D**) FoxP3-DTR mice were challenged with B16 melanoma tumors, similar to Fig. 1. After 5 days post-challenge, they were treated with diphtheria toxin (DT), with or without r3LCMV. (**E**) Tumor control. Data are pooled from 2 experiments (one experiment with n=5 per group and another with n=4-5 per group). Error bar represents SEM. Indicated P values were calculated by the Mann–Whitney test.

bioRxiv preprint doi: https://doi.org/10.1101/2023.12.08.570847; this version posted December 8, 2023. The copyright holder for this preprint (which was not certified by peer review) is the author/funder, who has granted bioRxiv a license to display the preprint in perpetuity. It is made available under aCC-BY 4.0 International license.

Figure S5

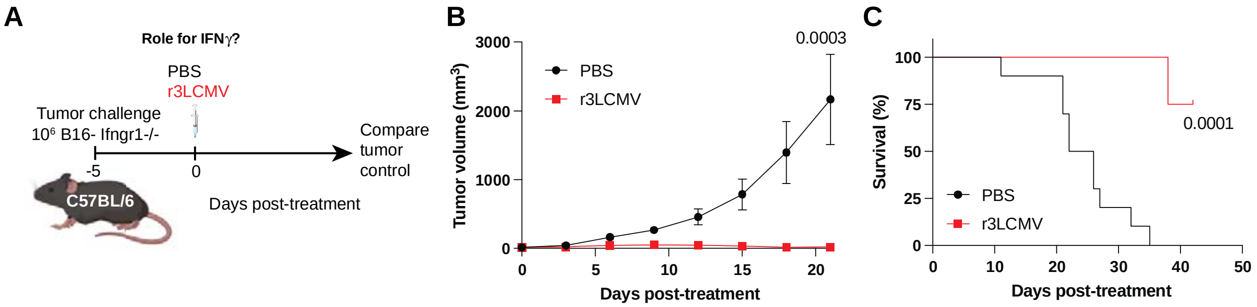

Figure S5. Tumor-intrinsic IFNγ signaling is not required for the antitumoral effect of r3LCMV. We tested the effect of r3LCMV vectors on B16 Ifngr1-/- melanoma. This tumor cannot sense IFNγ due to lack of its receptor. (**A**) Experiment outline. (**B**) Tumor control. (**C**) Survival. Data are from 1 experiment (n=8-9 per group). Error bar represents SEM. Indicated P values were calculated by the Mann–Whitney test, or Kaplan-Meier when comparing survival.

bioRxiv preprint doi: https://doi.org/10.1101/2023.12.08.570847; this version posted December 8, 2023. The copyright holder for this preprint (which was not certified by peer review) is the author/funder, who has granted bioRxiv a license to display the preprint in perpetuity. It is made available under aCC-BY 4.0 International license.

Figure S6

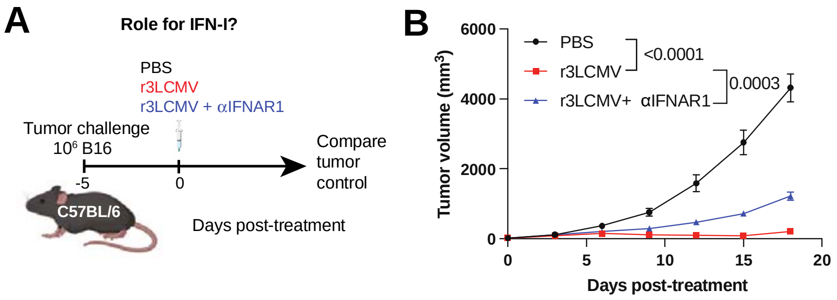

Figure S6. IFN-I signaling is partially required for the antitumoral effect of r3LCMV. We tested the effect of IFNAR1 blockade on r3LCMV therapy. (**A**) Experiment outline. (**B**) Tumor control. Data are pooled from 3 experiments (one experiment with n=6-7 per group, another with n=5, and and another with n=9-10 per group). Error bar represents SEM. Indicated P values were calculated using the Kruskal-Wallis test and Dunn's multiple comparisons test.

bioRxiv preprint doi: https://doi.org/10.1101/2023.12.08.570847; this version posted December 8, 2023. The copyright holder for this preprint (which was not certified by peer review) is the author/funder, who has granted bioRxiv a license to display the preprint in perpetuity. It is made available under aCC-BY 4.0 International license.

Figure S7

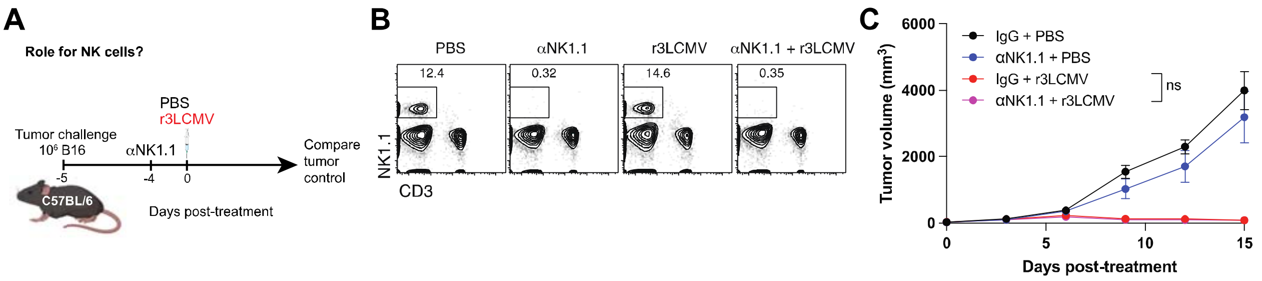

Figure S7. NK cells are not required for the antitumoral effect of r3LCMV. We tested the effect of NK cell depletion on r3LCMV therapy. (**A**) Experiment outline. (**B**) Representative FACS plots showing NK cells in PBMCs at day 0 of r3LCMV treatment (1 day after administration of αNK1.1). NK cell depleting antibodies (NK1.1, PK136) were administered at 500 μg, every 2 days, five times (see Materials and Methods). (**C**) Tumor control. Data are from 1 experiment (n=5 per group). Error bar represents SEM. Indicated P values were calculated using the Kruskal-Wallis test and Dunn's multiple comparisons test.

bioRxiv preprint doi: https://doi.org/10.1101/2023.12.08.570847; this version posted December 8, 2023. The copyright holder for this preprint (which was not certified by peer review) is the author/funder, who has granted bioRxiv a license to display the preprint in perpetuity. It is made available under aCC-BY 4.0 International license.

Figure S8

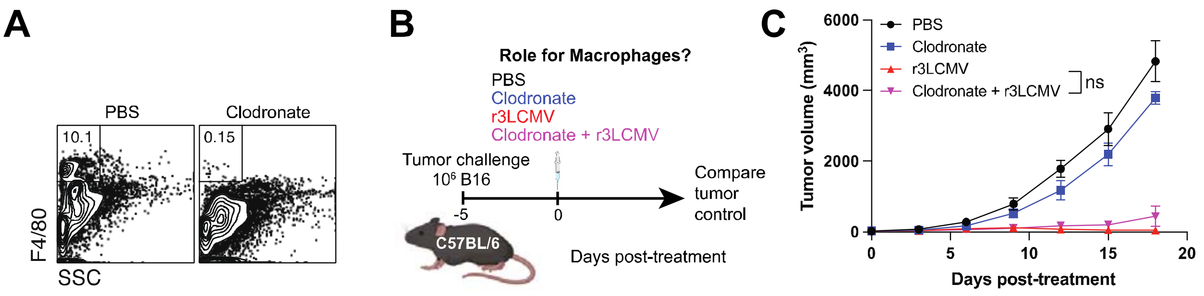

Figure S8. Macrophages are not required for the antitumoral effect of r3LCMV. We tested the effect of macrophage depletion on r3LCMV therapy. (**A**) Representative FACS plots of a pilot experiment showing macrophages in spleen at day 1 post-treatment (clodronate liposomes). This pilot showed that treatment with 200 µg of clodronate liposomes results in effective depletion of splenic macrophages. We used this same dose of clodronate liposomes. (**B**) Experiment outline. Clodronate liposomes were administered at 200 µg every 3 days, four times (see Materials and Methods). (**C**) Tumor control. Data are from 1 experiment (n=4-5 per group). Error bar represents SEM. Indicated P values were calculated using the Kruskal-Wallis test and Dunn's multiple comparisons test.

bioRxiv preprint doi: https://doi.org/10.1101/2023.12.08.570847; this version posted December 8, 2023. The copyright holder for this preprint (which was not certified by peer review) is the author/funder, who has granted bioRxiv a license to display the preprint in perpetuity. It is made available under aCC-BY 4.0 International license.

Figure S9

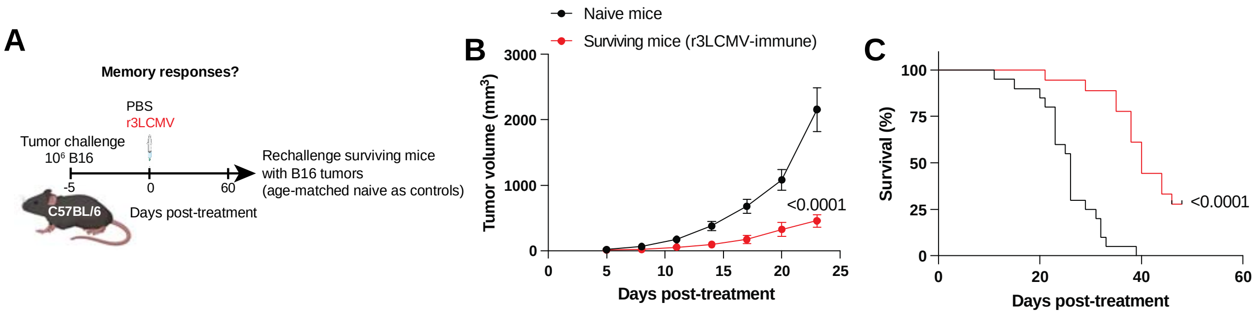

Figure S9. Immune memory after r3LCMV treatment. We tested whether mice that cleared tumors after r3LCMV therapy were protected upon subsequent tumor challenges. (**A**) Experiment outline. (**B**) Tumor control. (**C**) Survival. n=8-9 per group. Error bar represents SEM. Indicated P values were calculated using the Kruskal-Wallis test and Dunn's multiple comparisons test.
